# Quantitation of Six *Alternaria* Toxins in Infant Foods Applying Stable Isotope Labeled Standards

**DOI:** 10.3389/fmicb.2019.00109

**Published:** 2019-02-06

**Authors:** Marina Gotthardt, Stefan Asam, Klara Gunkel, Atefeh Fooladi Moghaddam, Elisabeth Baumann, Roland Kietz, Michael Rychlik

**Affiliations:** ^1^Chair of Analytical Food Chemistry, Technical University of Munich, Freising, Germany; ^2^National Nutrition and Food Technology Research Institute, Faculty of Nutrition Sciences and Food Technology, Shahid Beheshti University of Medical Sciences, Tehran, Iran

**Keywords:** *Alternaria* mycotoxins, stable isotope dilution assay (SIDA), LC-MS/MS, cereal based infant food, tomato products

## Abstract

*Alternaria* fungi are widely distributed saprophytes and plant pathogens. As pathogens, *Alternaria* fungi infect crops and vegetables and cause losses in the fields and during postharvest storage. While farmers suffer from declining yields, consumers are endangered by the formation of secondary metabolites, because some of these exhibit a pronounced toxicological potential. The evaluation of the toxicological capabilities is still ongoing and will contribute to a valid risk assessment. Additionally, data on the incidence and the quantity of *Alternaria* mycotoxins found in food products is necessary for dietary exposure evaluations. A sensitive LC-MS/MS method for the determination of the *Alternaria* mycotoxins alternariol (AOH), alternariol monomethylether (AME), tentoxin (TEN), altertoxin I (ATX I), alterperylenol (ALTP), and tenuazonic acid (TA) was developed. AOH, AME, and TA were quantified using stable-isotopically labeled standards. TEN, ATX I, and ALTP were determined using matrix matched calibration. The developed method was validated by using starch and fresh tomato matrix and resulted in limits of detection ranging from 0.05 to 1.25 μg/kg for starch (as a model for cereals) and from 0.01 to 1.36 μg/kg for fresh tomatoes. Limits of quantification were determined between 0.16 and 4.13 μg/kg for starch and between 0.02 and 5.56 μg/kg for tomatoes. Recoveries varied between 83 and 108% for starch and between 95 and 111% for tomatoes. Intra-day precisions were below 4% and inter-day precisions varied from 3 to 8% in both matrices. Various cereal based infant foods, jars containing vegetables and fruits as well as tomato products for infants were analyzed for *Alternaria* mycotoxin contamination (*n* = 25). TA was the most frequently determined mycotoxin and was detected in much higher contents than the other toxins. AME and TEN were quantified in many samples, but in low concentrations, whereas AOH, ATX I, and ALTP were determined rarely, among which AOH had higher concentration. Some infant food products were highly contaminated with *Alternaria* mycotoxins and the consumption of these individual products might pose a risk to the health of infants. However, when the mean or median is considered, no toxicological risk was obvious.

## Introduction

Fungi of the genus *Alternaria* are ubiquitous microorganisms growing on a wide range of substrates including soil, wall papers, decaying organic material and, most important from both toxicological and economical aspects, agricultural crops used for human and animal nutrition (Ostry, 2008). Infection of plants with *Alternaria* is commonly believed to occur on the field and many *Alternaria* species are well-known plant pathogens responsible for a series of plant diseases, e.g., black rot of tomatoes, black and gray rot of citrus fruits and black point of cereals (Logrieco et al., 2009). However, some *Alternaria* species are also able to grow at low temperature and are responsible for the postharvest decay of fruits and vegetables even at refrigerated storage or transport (Ozcelik et al., 1990). Unrevealing the taxonomy of the genus *Alternaria* is still a matter of ongoing research. Species differentiation by molecular biology seems to be more promising than the traditional morphologic approach (Zwickel et al., 2018).

**FIGURE 1:**
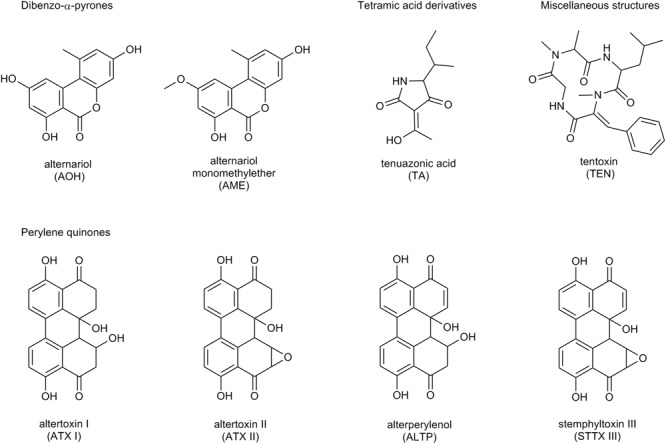
Structure of the *Alternaria* mycotoxins AOH, AME, TA, TEN, ATX I and II, ALTP, and STTX III.

The number of fungal secondary metabolites with toxic impact, the so-called mycotoxins, isolated from *Alternaria* fungi has reached at least 70 compounds up to now (Arcella et al., 2016). They exhibit great structural divergence and are commonly divided into five groups (Figure 1):

• Dibenzo-α-pyrones (e.g., alternariol, AOH; alternariol-monomethylether, AME),
• Tetramic acid derivatives (e.g., tenuazonic acid, TA),
• Perylene quinones (e.g., altertoxins I – III, ATX I – III),
• Specific toxins produced by *Alternaria alternata* subspecies *lycopersici* (AAL-toxins),
• Miscellaneous structures (e.g., tentoxin, TEN).

The variety of secondary fungal metabolites is further increased by the so-called modified mycotoxins (Rychlik et al., 2014). In case of *Alternaria* toxins the sulfate (S) conjugates AOH-3-S, AOH-9-S, and AME-3-S were identified as metabolites produced by the fungus itself, whereas the glucose (Glc) conjugates AOH-3-Glc, AOH-9-Glc, AME-3-Glc, and AME-7-Glc are regarded as plant metabolites (Soukup et al., 2016). Recently, AOH-9-Glc, AOH-3-S, and AME-3-S have been found for the first time in naturally contaminated tomato sauce for human consumption (Puntscher et al., 2018).

Analytical methods capable to determine this variety of analytes in food commodities are nowadays exclusively based on liquid chromatography coupled to tandem mass spectrometry (LC-MS/MS). In the recent years, methods have been developed to analyze three (Rodríguez-Carrasco et al., 2016), four (Berardis et al., 2018), five (Prelle et al., 2013), six (Noser et al., 2011), eight (Malachová et al., 2014), and even twelve (Zwickel et al., 2016) *Alternaria* toxins simultaneously, sometimes also including their modified forms (Walravens et al., 2014, 2016; Puntscher et al., 2018).

However, precise quantitation of these different substances still remains challenging, especially in multi-analyte approaches. Stable isotope dilution assays are meanwhile regarded as the gold standard of quantitative analytical methods and are more and more applied also in mycotoxin analysis (Asam and Rychlik, 2015). LC-MS/MS is severely affected by matrix interferences that can manifest themselves either as signal suppression or signal enhancement. By using stable isotope labeled internal standards, these interferences can be optimally minimized. Moreover, losses during sample preparation are also completely compensated for, because respective labeled standards and analytes are chemically indistinguishable and, therefore, show the same recovery throughout all steps of the sample preparation. In case of *Alternaria* mycotoxins the availability of labeled standards is still limited. So far, only the chemical syntheses of labeled AOH and AME (Asam et al., 2009; Liu and Rychlik, 2015), TEN (Liu and Rychlik, 2013), and TA (Asam et al., 2011b; Lohrey et al., 2013) and the biochemical isolation of labeled AOH, AME, altenuene (ALT), altenuisol (AS), ATX I – III, ALTP, and stemphyltoxin III (STTX III) (Liu and Rychlik, 2015) have been described.

The toxicity of *Alternaria* toxins has not yet been clarified in detail for all substances and is still a matter of ongoing research. AOH and AME are believed to be mutagenic because of their genotoxic effects *in vitro* (Brugger et al., 2006). On a molecular basis, AOH has been shown to interact with topoisomerases I and II with effects that have been described as “poisoning” (Fehr et al., 2009). Further research revealed that besides AOH, also AME, ATX I, ATX II, and ALTP are able to provoke these effects (Jarolim et al., 2016). Mutagenicity of ATX I, II, and III has long been known (Stack and Prival, 1986), but recently ATX II was identified as the major mutagen produced by *Alternaria* (Fleck et al., 2012; Schwarz et al., 2012), although the mode of mutagenic action of the latter is not clear yet (Fleck et al., 2016). TA is an acutely toxic substance with oral LD_50_ values between 81 and 225 mg/kg body weight for mice (Miller et al., 1963; Smith et al., 1968). TA inhibits protein biosynthesis by suppressing the release of new proteins from the ribosome (Shigeura and Gordon, 1963). Potentially, it is produced as virulence factor to facilitate colonization of the fungus on plants (Kang et al., 2017). TEN is considered as a phytotoxin, inhibiting photophosphorylation, and inducing chlorosis (Arntzen, 1972).

The Panel on Contaminants in the Food Chain (CONTAM) of the European Food Safety Authority (EFSA) evaluated the risks for public health related to *Alternaria* toxins in food in the year 2011 (EFSA, 2011). Their assessment was based on the threshold of toxicological concern (TTC) concept (Kroes et al., 2004) due to limited toxicity data for the *Alternaria* toxins. Although the panel also faced limited occurrence data, they estimated the critical TTC values to be exceeded by AOH and AME, but not by TEN and TA. In 2016, the EFSA reported a dietary exposure assessment of *Alternaria* toxins (Arcella et al., 2016). Compared to 2011, similar (AOH) or higher (AME, TEN, TA) exposures were estimated. Cereals and tomato based products were the main origin of *Alternaria* toxin intake and it was noted that infants were the population group with the highest dietary exposure.

In the year 2012, we conducted a survey about the contamination of infant food with the *Alternaria* mycotoxin TA. At this time, we found extremely high contamination of infant food samples based on millet (Asam and Rychlik, 2013). After toxicological evaluation, infant foods were regarded as a potential health hazard if a warning limit of 500 μg/kg of TA was exceeded (Rychlik et al., 2016). Few other studies dealt with this topic so far. In one study from Canada, AOH and AME were detected in 27 out of 30 samples of cereal-based infant food with maximum values of 4.4 μg/kg (AOH) and 9.0 μg/kg (AME) (Scott et al., 2012).

However, in our study in 2012 we were not able to include the other *Alternaria* toxins into the analytical scope due to limited sensitivity of the LC-MS equipment. Therefore, the aim of the present study was the development of a new method for the six *Alternaria* toxins AOH, AME, TA, TEN, ATX I, and ALTP and to apply this method to infant food in order to gain further insight in the actual contamination situation of infant food with *Alternaria* toxins.

## Materials and Methods

### Chemicals and Reagents

Analytical standards of AOH, AME, TA, and TEN were obtained from Sigma-Aldrich (Steinheim, Germany). ATX I, ATX II, ALTP, and STTX III were biosynthesized, purified by preparative HPLC and characterized by mass spectrometry (MS) and nuclear resonance spectroscopy (NMR) as reported earlier (Liu and Rychlik, 2013). TA was released from its commercial copper salt as described in the literature (Shephard et al., 1991; Siegel et al., 2009). The stable isotopically labeled standards [^2^H_4_]-AOH and [^2^H_4_]-AME were synthesized according to the literature (Asam et al., 2009). [^13^C_6_,^15^N]-TA was prepared in our laboratory as published (Asam et al., 2011b). The labeled standards were also chromatographically purified and characterized by MS and NMR studies.

Formic acid (≥98%) was obtained from Sigma-Aldrich (Steinheim, Germany). Ammonium formate for mass spectrometry (≥99.0%) was purchased from Sigma-Aldrich (Bellefonte, PA, United States). Ammonia solution (25%, for LC-MS) and starch (high purity) were obtained from Merck KGaA (Darmstadt, Germany). Acetonitrile (analytical grade), methanol (analytical grade), water (analytical and LC-MS grade), and isopropanol (LC-MS grade) were received from VWR International GmbH (Ismaning, Germany). Acetonitrile (LC-MS grade) was obtained from Carl Roth GmbH & Co., KG (Karlsruhe, Germany). Methanol (LC-MS grade) was purchased from Honeywell^TM^ Riedel-de Häen^TM^ (Seelze, Germany).

### Preparation of Stock Solutions and Calibration Standards

Stock solutions of unlabeled and labeled *Alternaria* mycotoxins were prepared in concentrations ranging from 10 to 100 μg/mL in acetonitrile (AOH, AME, and TEN) or methanol (ATX I, ALTP, and TA). The stock solutions were further diluted for sample preparations and method validations. All solutions were stored at -20°C in the dark. The concentrations of solutions were confirmed by UV spectrometry (Genesys, 10S, UV–Vis spectrophotometer, Thermo Fisher Scientific, Madison, WI, United States) using published extinction coefficients (Zwickel et al., 2016). However, ATX II and STTX III were available only in amounts that were not detectable by UV–VIS and, therefore, were only qualitatively included in the method.

### LC-MS/MS Analysis

The chromatographic separation of the analytes was performed on a Shimadzu Nexera X2 UHPLC system (Shimadzu, Kyoto, Japan). The LC-parameters for AOH, AME, TEN, ATX I, ATX II, STTX III, and ALTP were adopted from Liu and Rychlik (2013, 2015). A HyperClone BDS-C18 column (150 × 3.2 mm, 3 μm, 130 Å, Phenomenex, Aschaffenburg, Germany) protected by a C18-guard column (4 × 2.0 mm ID, Phenomenex, Aschaffenburg, Germany) served as stationary phase. The column was tempered to 30°C. The flow rate was set to 0.2 mL/min. The binary gradient system consisted of acetonitrile/2-propanol/water (solvent A, 17.5/17.5/65, *v*/*v*/*v*) and methanol (solvent B). The mobile phase was held at 0% B for the first 3 min. The gradient raised linearly from 0 to 100% B in the following 19 min and remained at 100% B for 1 further min. The mobile phase returned to 0% B within the next 2 min and the column was equilibrated for 5 min. 10 μL were used as injection volume. Using an additional valve after the column, the flow was introduced into the mass spectrometer not until 6.9 min after injection to prevent the instrument from matrix contamination.

Due to the different polarity of TA, the mycotoxin had to be analyzed in an additional LC-MS/MS run. The LC-parameters for TA were based on Asam et al. (2013). A Gemini-NX C18 column (150 × 4.6 mm, 3 μm, 110 Å, Phenomenex, Aschaffenburg, Germany) protected by a Gemini-NX C18-guard column (4 × 3.0 mm ID, Phenomenex, Aschaffenburg, Germany) served as stationary phase. The column was tempered to 40°C. The flow rate was set to 0.5 mL/min. The binary gradient system consisted of 5 mM ammonium formate (solvent A, pH 9) and methanol (solvent B). The mobile phase was held at 5% B for 3 min, raised linearly from 5 to 100% B in 5 min and remained at 100% B for 2 min. Thereafter, the gradient returned to 5% B within 3 min and the column was equilibrated for 10 min. The injection volume was 10 μL. Using an additional valve after the column, the flow was introduced into the mass spectrometer not until 6.5 min after injection to prevent the instrument from matrix contamination.

Using automated column switching and fourfold solvent selection provided by the instrument, both methods could be run in the same sequence.

The LC was coupled to a triple quadrupole ion trap mass spectrometer (LCMS-8050, Shimadzu Corporation, Kyoto, Japan). The instrument operated in the negative electrospray ionization (ESI) mode for all analytes. Parameters for the interface were adjusted as follows: heating gas flow 10 L/min, nebulizing gas flow 3 L/min, drying gas flow 10 L/min, heat block temperature 400°C, desolvation line temperature 250°C, interface temperature 300°C, interface voltage 4 kV and collision-induced dissociation gas pressure 270 kPa. The mass spectrometer worked in the scheduled multiple reaction monitoring (MRM) mode for MS/MS measurements. The voltages for the fragmentation of each analyte were optimized via direct infusion of standard solutions of AOH, AME, TEN, ATX I, ATX II, ALTP, STTX III, and TA at a concentration of 1 μg/mL. Two mass transitions were chosen, one as quantifier and one as qualifier for confirmation. Optimized voltages and collision energies, the retention time as well as the quantifier/qualifier ratio of each analyte are listed in Table 1. The LabSolutions software (Shimadzu, Kyoto, Japan) was utilized for data acquisition and data analysis.

**Table 1:** Precursor ions and product ions of the unlabeled and labeled *Alternaria* mycotoxins, optimized fragmentation conditions, retention times, and quantifier/qualifier ratio.

Analyte	Precursor ion *m/z*	Product ion *m/z*	Q1 Pre-bias [V]	CE [V]	Q3 Pre-bias [V]	Retention time [min]	Ratio quantifier/qualifier^∗^
ATX I	351.20	297.20	25	28	19	9.6	1.15
		314.20	27	32	21		
ALTP	349.30	261.25	18	28	15	10.2	0.74
		303.00	18	19	18		
AOH	257.30	213.00	15	23	12	11.4	1.27
		215.05	14	24	13		
[^2^H_4_]-AOH	261.30	217.00	15	23	12	11.4	2.09
		218.05	14	24	13		
TEN	413.30	141.05	15	23	23	12.5	1.46
		271.30	15	17	17		
ATX II	349.40	313.25	16	25	12	13.6	0.45
		331.30	20	15	20		
STTX III	347.20	329.20	17	22	21	15.4	1.16
		301.10	17	35	18		
AME	271.30	256.20	20	23	25	18.2	3.63
		228.20	19	30	13		
[^2^H_4_]-AME	275.30	260.20	20	23	25	18.2	3.47
		232.20	19	30	13		
TA	196.30	112.05	22	26	20	8.3	1.01
		139.00	14	22	11		
[^13^C_6,15_N]-TA	203.25	113.05	22	26	20	8.3	0.91
		142.00	14	22	11		

*^∗^Mean of five injections. The quantifier/qualifier ratios were determined in pure solvents.*

### Sample Preparation

In preliminary experiments, different solvent mixtures consisting of acetonitrile, methanol, and water were tested for ideal mycotoxin extraction from grain matrix. The extraction was optimized by adding different amounts of formic acid and acetic acid as well as the adjustment of the extraction duration. Various cartridges with different sorbent material (hydrophilic-lipophilic-balanced (HLB, Waters Corporation, Milford, MA, United States), C18 (Discovery^®^ DSC-18, Supelco, Bellefonte, PA, United States), polyamide (PA, CHROMABOND^®^, Machery-Nagel GmbH & Co., KG, Düren, Germany), NH_2_ (CHROMABOND^®^, Machery-Nagel GmbH & Co., KG, Düren, Germany), and Strata-X (Polymeric reversed phase, Phenomenex, Aschaffenburg, Germany) were tested for the simultaneous purification of *Alternaria* mycotoxins with special regards to TA. From the results of these experiments the optimized sample preparation method was developed as follows:

One gram of ground and homogenized sample was weighed into a 50 mL centrifuge tube and the isotopically labeled internal standards were added to the sample (100 μL of [^2^H_4_]-AOH (0.1 μg/mL), 50 μL of [^2^H_4_]-AME (0.1 μg/mL) and 100 μL of [^13^C_6_,^15^N]-TA (1.0 μg/mL)). The mycotoxins were extracted by adding 15 mL of a mixture of acetonitrile/water (84/16; *v*/*v*) and 0.2 mL of formic acid and horizontal shaking for 30 min (175 rpm). The sample was centrifuged at room temperature at 1690 × g for 5 min and the residue was extracted again as described above. The third extraction step was performed by adding 15 mL of a mixture of acetonitrile/methanol/water (50/25/25; *v*/*v*/*v*) and 0.3 mL of formic acid. After 30 min of horizontal shaking (175 rpm), the sample was filtered. The combined extracts and the filtrate were rotary evaporated to dryness at 40°C. The residue was reconstituted in 12 mL of water (pH 5.5). The mycotoxins were purified by solid phase extraction (Discovery^®^ DSC-C18, 500 mg, 6 mL, Sigma-Aldrich, Bellefonte, PA, United States). The C18 material was washed with 6 mL of methanol and conditioned with 6 mL of water (pH 5.5). The sample was completely loaded onto the column. After washing the column with 12 mL of water, the column was dried under vacuum. The mycotoxins were eluted with 6 mL of methanol. For TA, an additional elution step with 9 mL of methanol + 2% ammonium hydroxide was performed. The eluate was rotary evaporated to dryness at 40°C. The residue was reconstituted in 1 mL of methanol/water (1/1; *v*/*v*). Remaining matrix precipitated during night at -20°C. The sample was membrane filtered (0.22 μm) and analyzed by LC-MS/MS.

Twenty-five infant food samples were collected from various supermarkets and drugstores in Germany. Cereal based infant food samples were either single grain or multi-grain products, containing spelt, oat, millet, rice, and wheat. Jars, containing either vegetables or fruits as well as two tomato products were purchased.

### Calibration and Quantitation

AOH, AME, and TA were quantified using isotopically labeled internal standards, whereas TEN, ATX I, and ALTP were quantified using matrix matched calibration.

For AOH, AME, and TA, calibration functions were obtained by mixing various amounts of analyte (A) with constant amounts of internal standard (IS). The calibration functions ranged from molar ratios [n(A)/n(IS)] of 0.01 to 100 (1:100, 1:50, 1:10, 1:5, 1:2, 1:1, 2:1, 5:1, 10:1, 100:1). The internal standards for AOH and AME were [^2^H_4_]-labeled. For TA, the [^13^C_6_,^15^N]-labeled isotopologue served as internal standard. The calibration points were measured by LC-MS/MS. The calibration functions were calculated using linear regression after plotting molar ratios [n(A)/n(IS)] against peak area ratios [A(A)/A(IS)]. Linearity was confirmed by Mandel’s fitting test.

Matrix matched calibrations for TEN, ATX I, and ALTP were performed on two different matrices. Potato starch was spiked with various amounts of TEN (0.1–100 μg/kg), ATX I (0.4–20 μg/kg), and ALTP (0.6–20 μg/kg). Blank tomato puree was spiked with varying amounts of TEN (0.1–100 μg/kg), ATX I (0.4–20 μg/kg), and ALTP (0.4–20 μg/kg). After sample preparation, the calibration points were analyzed by LC-MS/MS. The calibration curve was constructed from peak area [A(A)] against spiked contents of the analyte [w(A)] and the calibration function was received via linear regression. Linearity of the matrix matched calibration functions was confirmed by Mandel’s fitting test.

Mycotoxin contamination in cereal based infant food samples or jars was either calculated by using the respective calibration function (AOH, AME, and TA) or by using the matrix-matched calibration function (TEN, ATX I, and ALTP).

### Method Validation

The sample preparation and the LC-MS/MS method were validated according to Vogelgesang and Hädrich (1998). Potato starch was chosen as surrogate blank matrix for the method validation, because all analyzed grain flours contained *Alternaria* mycotoxins. To obtain a blank tomato matrix, fresh and sound whole tomatoes were pureed and analyzed by LC-MS/MS to confirm the absence of *Alternaria* mycotoxins.

For the determination of limits of detection (LODs) and quantitation (LOQs), AOH, AME, TEN, ATX I, ALTP, and TA were spiked to the blank matrices at four different levels, respectively. Potato starch was spiked with AOH (1.0, 3.0, 7.0, and 10 μg/kg), AME (0.1, 0.3, 0.7, and 1.0 μg/kg), TEN (0.1, 0.4, 0.7, and 1.0 μg/kg), ATX I (0.8, 2.0, 5.0, and 8.0 μg/kg), ALTP (0.4, 1.6, 2.8, and 4.0 μg/kg), and TA (2.5, 8.0, 16, and 25 μg/kg). Fresh tomatoes were spiked with AOH (0.15, 0.6, 1.0, and 1.5 μg/kg), AME (0.015, 0.06, 0.1, and 0.15 μg/kg), TEN (0.15, 0.6, 1.0, and 1.5 μg/kg), ATX I (0.5, 2.0, 3.5, and 5.0 μg/kg), ALTP (0.4, 1.6, 2.8, and 4.0 μg/kg), and TA (1.5, 6.0, 10, and 15 μg/kg). Each level was prepared in triplicate. The sample preparation was performed as described above and samples were analyzed by LC-MS/MS.

The recoveries for each mycotoxin were determined in starch and tomato matrix at three different levels, respectively. Potato starch was spiked with AOH (3.0, 7.0, and 10 μg/kg), AME (0.3, 0.7, and 1.0 μg/kg), TEN (0.4, 0.7, and 1.0 μg/kg), ATX I (2.0, 5.0, and 8.0 μg/kg), ALTP (1.6, 2.8, and 4.0 μg/kg), and TA (15, 100, and 200 μg/kg). Tomato puree was spiked with AOH (0.6, 1.0, and 1.5 μg/kg), AME (0.06, 0.1, and 0.15 μg/kg), TEN (0.6, 1.0, and 1.5 μg/kg), ATX I (2.0, 3.5, and 5.0 μg/kg), ALTP (1.6, 2.8, and 4.0 μg/kg), and TA (50, 100, and 200 μg/kg). Each level was prepared in triplicate. The samples were prepared as described above and analyzed by LC-MS/MS. The content of toxins in the samples were calculated either using SIDA or matrix matched calibration and the recovery was calculated for each toxin as follows: R = found amount [μg/kg]/spiked amount [μg/kg].

Infant food samples containing *Alternaria* mycotoxins were analyzed to determine the inter-injection precisions, intra- and inter-day precisions. A seven-grain infant food product contained TEN (23 μg/kg), ALTP (6 μg/kg), and TA (114 μg/kg) and was spiked with AOH (10 μg/kg), AME (1.5 μg/kg), and ATX I (6 μg/kg). An organic tomato sauce contained AOH (75 μg/kg), AME (9 μg/kg), and TA (490 μg/kg) and was spiked with TEN (8 μg/kg), ATX I (5 μg/kg), and ALTP (6 μg/kg).

The inter-injection precision was determined by multiple injections of one sample (*n* = 5). The intra-day precision was calculated after injecting three samples in triplicate into the LC-MS/MS. For the inter-day precision, the samples were prepared in triplicate in 3 weeks and injected in triplicate into the LC-MS/MS. The mycotoxin contamination was either calculated by using the respective calibration function (AOH, AME, and TA) or by using the matrix-matched calibration function (TEN, ATX I, and ALTP).

## Results

### Method Development

The *Alternaria* mycotoxins AOH, AME, TEN, ATX I, ALTP, and TA were extracted from food samples, purified by solid phase extraction and analyzed by LC-MS/MS. To optimize the extraction of *Alternaria* mycotoxins from grain matrix, different mixtures of acetonitrile, methanol, and water were tested. Applying only one extraction step resulted in insufficient recoveries of the mycotoxins. Therefore, two additional extraction steps were indispensable to extract the mycotoxins sufficiently. For the first and second extraction, a mixture of acetonitrile/water (84/16; *v*/*v*) and the addition of formic acid showed best extraction sufficiency for most of the mycotoxins. The volume of the extraction solvents was set to 15 mL, on the one hand, to obtain good recoveries and, on the other hand, to save time during evaporation. Especially the extraction of the perylene quinones required a third extraction step. The addition of methanol to the extraction solvent increased the recoveries of the perylene quinones and the solvent mixture was optimized to acetonitrile/methanol/water (50/25/25; *v*/*v*/*v*). The addition of formic acid to the extraction solvent improved in particular the extraction efficiency of the perylene quinones. The mycotoxin extracts were then purified by solid phase extraction. Different solid phases were tested for their matrix reduction and their mycotoxin retention, in particular for their simultaneous retention of TA together with the other *Alternaria* mycotoxins. Promising results provided only C18, Strata-X and HLB cartridges as all tested mycotoxins were retained sufficiently by these sorbent materials. Due to economic reasons, C18 material was finally chosen as sorbent material for the sample preparation. The *Alternaria* mycotoxins show different polarity and, therefore, the pH during the conditioning, sample loading, and elution was optimized with special regards to TA. For optimal retention of TA on C18 during sample loading, the pH of the sample had to be adjusted to 5.5. Elution of the mycotoxins was performed using 6 mL of methanol. The recovery of TA was improved by applying a second elution step with methanol containing 2% of ammonium hydroxide. The amount of sample needed for analysis was also adjusted. Different amounts of sample were weighed into centrifuge tubes and the recovery of mycotoxins was determined. Best recoveries were obtained for 1 g of sample, as 1.5 or 2 g of sample needed to be extracted with more solvent. The usage of more solvent would also increase duration of evaporation and, therefore, sample preparation time. Less than one gram of sample might not reflect inhomogeneity of the sample sufficiently. The developed sample preparation method for grains was totally applicable to tomato matrix.

### LC-MS/MS Analysis

**FIGURE 2:**
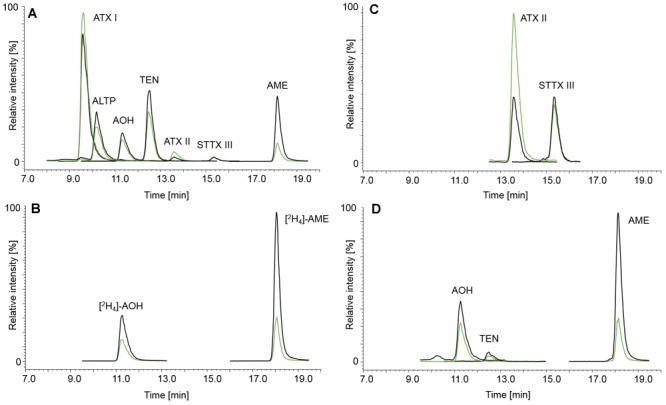
Chromatograms of the *Alternaria* mycotoxins ATX I (130 μg/kg), ALTP (20.0 μg/kg), AOH (20.0 μg/kg), TEN (25.0 μg/kg), ATX II (approximately 2.00 μg/kg, see Section “Preparation of Stock Solutions and Calibration Standards”), STTX III (approximately 3.00 μg/kg, see Section “Preparation of Stock Solutions and Calibration Standards”), and AME (5.00 μg/kg) **(A)** and the isotopically labeled internals standards [^2^H_4_]-AOH (20.0 μg/kg) and [^2^H_4_]-AME (5.00 μg/kg) **(B)**. Chromatogram **(C)** shows the standards ATX II (approximately 2.00 μg/kg, see Section “Preparation of Stock Solutions and Calibration Standards”) and STTX III (approximately 3.00 μg/kg, see Section “Preparation of Stock Solutions and Calibration Standards”) in more detail. The mycotoxin contamination of an infant food in jar containing apple, pear, and cherry is depicted in chromatogram **(D)**, containing AOH (2.86 μg/kg), TEN (0.93 μg/kg), and AME (1.42 μg/kg). The black and green mass transitions represent the quantifier and qualifier of the analyte and labeled internal standard, respectively.

**FIGURE 3:**
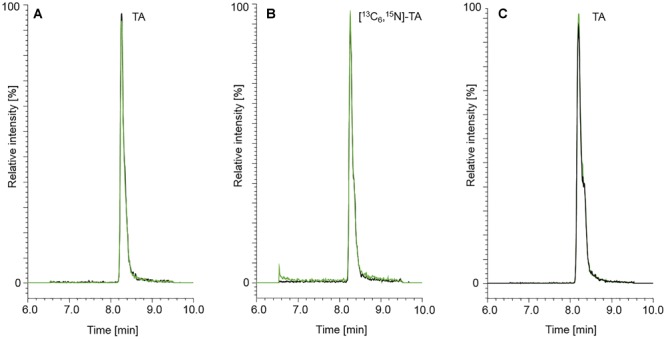
Chromatograms of TA (50.0 μg/kg) **(A)** and the isotopically labeled internal standard [^13^C_6_,^15^N]-TA (50.0 μg/kg) **(B)**. A naturally contaminated seven-grains infant food sample is shown in chromatogram **(C)**. The content of TA determined in the seven-grains infant food was 149 μg/kg. The black and green mass transitions represent the quantifier and qualifier of the analyte and labeled internal standard, respectively.

**FIGURE 4:**
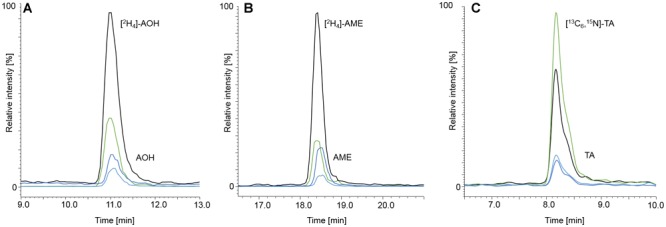
Chromatograms at LOQ level of AOH, [^2^H_4_]-AOH **(A)**, AME, [^2^H_4_]-AME **(B)**, TA and [^13^C_6_,^15^N]-TA **(C)**. AOH, AME, and TA are depicted in spiked starch matrix at contents of 1.00, 0.10, and 2.50 μg/kg, respectively. The labeled internal standards were added to the matrix at contents of 10.0 μg/kg for [^2^H_4_]-AOH, 5.00 μg/kg for [^2^H_4_]-AME, and 10.0 μg/kg for [^13^C_6_,^15^N]-TA. The black and the green mass transitions represent the quantifiers and the qualifiers of the labeled standards, whereas the dark blue and light blue mass transitions represent the quantifiers and qualifiers of the analytes, respectively.

The LC-parameters for AOH, AME, TEN, ATX I, ATX II, STTX III, and ALTP were adopted from Liu and Rychlik (2013, 2015). Figure 2 displays LC-MS/MS chromatograms of ATX I, ALTP, AOH, TEN, and AME **(A)**, of the internal standards [^2^H_4_]-AOH and [^2^H_4_]-AME **(B)**, and of ATX II and STTX III **(C)**. A naturally contaminated infant food in jar, consisting of apple, pear, and cherry, is shown in chromatogram **(D)**. TA was added to the existing chromatographic run but due to different polarity, the retention was unsatisfyingly low. Therefore, TA had to be analyzed in a separate LC-MS/MS run. The LC-parameters for TA were based on Asam et al. (2013). Chromatograms of TA **(A)** and [^13^C_6_,^15^N]-TA **(B)** are displayed in Figure 3. Chromatogram **(C)** shows a naturally contaminated seven-grain infant food sample. In Figure 4, AOH, AME, and TA are depicted in contents of 1.00, 0.10, and 2.50 μg/kg. The labeled internal standards [^2^H_4_]-AOH, [^2^H_4_]-AME, and [^13^C_6_,^15^N]-TA are shown at contents of 10.0, 5.00, and 10.0 μg/kg. Due to the isotope effect, slight retention time shifts of the deuterated internal standards [^2^H_4_]-AOH and [^2^H_4_]-AME compared to the unlabeled analytes AOH and AME are observed during the chromatographic separation. The mass difference between [^13^C] and [^12^C] is proportionally much lower than between [^2^H] and [^1^H] and, therefore, the retention time shift between [^13^C_6_,^15^N]-TA and TA is not as pronounced as for the deuterated standards (Rychlik and Asam, 2008).

The fragmentation of AOH, AME, TEN, ATX I, ATX II, STTX III, ALTP, and TA was optimized by injecting solutions of reference compounds (1.0 μg/mL). Mass transitions of the quantifiers and qualifiers of the analytes were checked for interfering matrix compounds. As the matrix did not overlap with the mass transitions of the analytes, specificity of AOH, AME, TEN, ATX I, ALTP, and TA was assumed during analysis, but constantly monitored through the quantifier/qualifier ratio.

### Calibration and Quantification

Calibration functions of AOH, AME, and TA were obtained by analyzing mixtures of analyte with the isotopically labeled internal standards. Linearity was confirmed between molar ratios of analyte (A) and internal standard (IS) [n(A)/n(IS)] of 0.01 to 100, respectively.

Matrix matched calibrations for TEN, ATX I, and ALTP were performed for two different matrices to examine the range of linearity. The linearity of the matrix matched calibration functions was confirmed from 0.1 to 100 μg/kg for TEN, from 0.4 to 20 μg/kg for ATX I, and from 0.6 to 20 μg/kg for ALTP in starch matrix. In tomato puree, the linearity of the calibration functions was confirmed from 0.1 to 100 μg/kg for TEN, from 0.4 to 20 μg/kg for ATX I, and from 0.4 to 20 μg/kg for ALTP.

### Method Validation

Limits of detection (LOD) and limits of quantification (LOQ) were determined according to Vogelgesang and Hädrich (1998). As no cereal flour from the supermarket was free from *Alternaria* mycotoxins, potato starch was chosen as blank matrix for the method validation. To represent the jar’s matrix and the tomato products, fresh tomato puree was chosen as blank matrix for the method validation. AOH, AME, TEN, ATX I, ALTP, and TA were spiked to the blank matrices in triplicate at four different spiking levels. LODs ranged from 0.05 to 1.25 μg/kg in starch matrix (Table 2), and from 0.01 to 1.36 μg/kg in tomato matrix (Table 3). LOQs ranged from 0.16 to 4.13 μg/kg in starch and from 0.02 to 5.56 μg/kg in tomato puree (Tables 2,3).

**Table 2:** Limits of detection, limits of quantification, recoveries, and precisions determined for *Alternaria* mycotoxins in starch.

Analyte	LOD [μg/kg]	LOQ [μg/kg]	Recovery [%]			Precision (RSD) [%]		
			Level 1	Level 2	Level 3	Inter-injection (*n* = 5)	Intra-day (*n* = 3)	Inter-day (*n* = 9)
AOH	0.50	1.81	101 ± 6	97 ± 2	92 ± 2	2	2	5
AME	0.05	0.23	94 ± 8	103 ± 1	108 ± 5	2	2	3
TEN	0.05	0.16	91 ± 7	83 ± 1	83 ± 2	3	3	7
ATX I	0.42	1.49	101 ± 6	94 ± 5	91 ± 3	2	3	6
ALTP	0.31	1.03	105 ± 5	94 ± 2	97 ± 4	2	1	5
TA	1.25	4.13	95 ± 2	92 ± 5	106 ± 4	2	1	5

**Table 3:** Limits of detection, limits of quantification, recoveries, and precisions determined for *Alternaria* mycotoxins in fresh tomatoes.

Analyte	LOD [μg/kg]	LOQ [μg/kg]	Recovery [%]			Precision (RSD) [%]		
			Level 1	Level 2	Level 3	Inter-injection (*n* = 5)	Intra-day (*n* = 3)	Inter-day (*n* = 9)
AOH	0.16	0.55	103 ± 3	95 ± 9	100 ± 2	1	1	3
AME	0.01	0.02	111 ± 1	97 ± 1	96 ± 2	1	1	4
TEN	0.05	0.22	101 ± 3	107 ± 2	109 ± 1	1	1	6
ATX I	0.19	0.76	107 ± 4	101 ± 2	103 ± 2	2	1	4
ALTP	0.21	0.74	107 ± 4	97 ± 2	100 ± 1	1	3	5
TA	1.36	5.56	107 ± 2	99 ± 1	96 ± 3	2	1	8

Recoveries were determined by spiking AOH, AME, TEN, ATX I, ALTP, and TA to the respective blank matrices. The level of spiking complied with contents of the mycotoxins determined in naturally contaminated food products. For the starch matrix, the recoveries ranged from 83 to 108% (Table 2). For tomato matrix, the recoveries ranged from 95 to 111% (Table 3).

Various precisions (relative standard deviations) of the analytes were calculated (Tables 2,3). Inter-injection precisions were determined by repetitive injecting (*n* = 5) a cereal based infant food sample or the tomato soup into the LC-MS/MS instrument. Relative standard deviations ranged from 2 to 3% in the cereal based product and from 1 to 2% in the tomato soup. The intra-day and inter-day precisions were determined by preparing one sample in triplicate and in 3 weeks. The calculated intra-day precisions lay between 1 and 3% in cereals and between 1 and 3% in the tomato matrix. Inter-day precisions varied from 3 to 7% in the cereal based infant food and from 3 to 8% in the tomato soup.

### Analysis of Infant Food Samples

Various cereal based infant food samples (*n* = 19) as well as jars (*n* = 6) were purchased from local drugstores and supermarkets and analyzed for their content of AOH, AME, TEN, ATX I, ALTP, and TA (Table 4). Within the cereal based infant foods, single grain as well as multi-grain products were analyzed. ATX II and STTX III were not detected in any of the infant food samples. The other *Alternaria* mycotoxins were detected in cereal based infant food above the limit of quantification in 17 out of 19 samples. TA was determined in 89% of the products with contents above the limit of quantification. The contamination of the latter ranged from 5.66 to 221 μg/kg and exceeded the contents of the other mycotoxins. Single grain products with partially high contamination of TA were millet (221 μg/kg), spelt (102 μg/kg), rice (109 μg/kg), and two multi-grain products (104 and 149 μg/kg). The median contamination of TA in the cereal based products was 22.0 μg/kg. AOH was determined in one spelt and one multi-grain product with contents of 7.17 and 4.73 μg/kg. AME was determined in 53% of all cereal based infant products with contents above the limit of quantification. The contamination of AME ranged from 0.23 to 0.58 μg/kg with a median of 0.23 μg/kg, which can be considered as low contamination. Eighty-four percent of all products contained TEN in amounts from 0.18 to 7.53 μg/kg. The median was 0.43 μg/kg. ATX I was detected in only one wheat infant food sample with a content of 1.52 μg/kg. ALTP was detected in one multi-grain product above the limit of quantification with a content of 4.47 μg/kg.

**Table 4:** Contents of *Alternaria* mycotoxins in infant foods based on the cereals indicated.

Variety	AOH [μg/kg]	AME [μg/kg]	TEN [μg/kg]	ATX I [μg/kg]	ALTP [μg/kg]	TA [μg/kg]
Wheat 1	–	(0.15) ^a^	–	–	–	(2.62) ^a^
Wheat 2	(0.76) ^a^	0.37 ± 0.13	0.60 ± 0.06	1.52 ± 0.11	–	8.38 ± 0.28
Wheat 3	–	0.33 ± 0.11	1.29 ± 0.09	–	–	10.2 ± 0.1
Wheat 4	–	–	(0.06)^a^	–	–	–
Oat 1	–	(0.08)^a^	(0.10)^a^	–	–	22.0 ± 0.5
Oat 2	–	0.25 ± 0.01	0.18 ± 0.02	–	–	8.54 ± 0.75
Millet 1	–	(0.06) ^a^	0.33 ± 0.04	–	–	43.6 ± 0.2
Millet 2	(1.01) ^a^	0.35 ± 0.05	0.86 ± 0.03	–	–	221 ± 2
Spelt 1	–	(0.05)^a^	0.43 ± 0.01	–	–	102 ± 1
Spelt 2	7.17 ± 0.65	0.27 ± 0.07	0.41 ± 0.15	(0.67) ^a^	–	49.6 ± 3.0
Spelt 3	(0.67) ^a^	(0.05)^a^	0.72 ± 0.08	–	–	23.7 ± 0.5
Rice 1	–	0.58 ± 0.24	2.22 ± 0.36	–	–	109 ± 1
rice 2	(0.67) ^a^	(0.13)^a^	0.30 ± 0.04	–	–	10.1 ± 0.1
Multi-grain 1	4.73 ± 0.04	0.55 ± 0.21	0.41 ± 0.03	–	–	12.4 ± 0.1
Multi-grain 2	–	–	0.21 ± 0.02	–	–	5.66 ± 0.31
Multi-grain 3	–	0.23 ± 0.06	0.62 ± 0.17	–	–	66.0 ± 0.9
Multi-grain 4	(1.37) ^a^	0.56 ± 0.13	0.60 ± 0.06	–	–	6.15 ± 0.49
Multi-grain 5	–	0.46 ± 0.08	2.08 ± 0.55	(0.81) ^a^	–	104 ± 1
Multi-grain 6	–	0.07 ^a^	7.53 ± 0.97	–	4.47 ± 0.31	149 ± 1
Mean^b^	0.89	0.24	1.00	0.17	0.24	50.2
Median^b^	0	0.23	0.43	0	0	22.0

*– : not detected, ^*a*^: value between LOD and LOQ, ^*b*^: calculation of mean and median: 0 μg/kg were used for “-”, the twofold value of the respective LODs were used for values below the LOQs.*

Four different purees in jars as well as one tomato sauce and one tomato soup were analyzed for AOH, AME, TEN, ATX I, ALTP, and TA contamination (Table 5). Five out of six samples contained *Alternaria* mycotoxins above the limit of quantification. AOH and TEN were determined in one jar with contents of 2.86 and 0.44 μg/kg. AME was determined in contents ranging from 0.04 to 7.56 μg/kg with a median of 0.14 μg/kg. TA was detected in two of the purees at a mean of 4.95 μg/kg. ATX I, ATX II, ALTP, and STTX III were not detected in any of the puree products. 8.73 μg/kg of TA were quantified in the tomato soup, whereas the tomato sauce contained high amounts of AOH (54.2 μg/kg), AME (7.56 μg/kg), and TA (505 μg/kg).

**Table 5:** Contents of *Alternaria* mycotoxins in puree infant food consisting of vegetables and fruits, and in tomato products.

Variety	AOH [μg/kg]	AME [μg/kg]	TEN [μg/kg]	ATX I [μg/kg]	ALTP [μg/kg]	TA [μg/kg]
Tomato sauce	54.2 ± 2.8	7.56 ± 0.72	0.44 ± 0.09	–	–	505 ± 26
Apple-pear-cherry	2.86 ± 0.99	1.42 ± 0.07	0.93 ± 0.02	–	–	(5.38) ^a^
Pumpkin and potato	(0.32) ^a^	–	–	–	–	–
Cherry and banana	(0.17) ^a^	0.14 ± 0.07	–	–	–	(4.51) ^a^
Vegetable	–	0.04 ± 0.02	–	–	–	–
Tomato soup	–	0.04 ± 0.01	–	–	–	8.73 ± 0.03
Mean^b^	9.62	1.53	0.23	–	–	86.5
Median^b^	0.32	0.14	0	–	–	2.51

*–: not detected, ^*a*^: value between LOD and LOQ, ^*b*^: calculation of mean and median: 0 μg/kg were used for “-”, the twofold value of the respective LODs were used for values below the LOQs.*

## Discussion

### Method Validation

LODs and LOQs determined in our method validation process are in accordance with the LODs and LOQs of methods recently reported in the literature. Liu et al. determined LODs and LOQs in starch matrix of 0.088 to 0.36 μg/kg and 0.27 to 1.1 μg/kg for AOH, AME, ATX I, and ALTP (Liu and Rychlik, 2015). For TA, low LODs and LOQs were achieved for both matrices. As the sample preparation and sample clean-up was extensively optimized for TA, derivatization with 2,4-dinitrophenylhydrazine (Siegel et al., 2009; Asam et al., 2011b) was not necessary to obtain low LODs and LOQs.

Zwickel et al. calculated LOQs of AOH, AME, TEN, ATX I, and TA of 0.4 up to 0.9 μg/kg in tomato juice (Zwickel et al., 2016). Puntscher et al. recently achieved LODs and LOQs in tomato sauce of 0.05–6 and 0.1–12 μg/kg with a dilute and shoot approach (Puntscher et al., 2018).

In our method validation process, recoveries ranged from 83 to 111% for AOH, AME, TEN, ATX I, ALTP, and TA in both matrices. To achieve these good recoveries, the samples needed to be extracted three times with different solvent mixtures. Performing only one extraction step resulted in losses of analytes between 19 and 42%. AOH, AME, and TA were quantified using stable isotopically labeled internal standards and the recoveries ranged from 92 to 111%. Quantification of TEN, ATX I, and ALTP by matrix matched calibrations resulted in a slightly lower recovery rate for TEN at two spiking levels. For ATX I and ALTP, matrix matched calibrations compensated for analyte losses during sample preparation and recoveries varied between 91 and 109%.

Inter-injection, intra- and inter-day precisions were determined for cereal and tomato based products and resulted in precisions below 4% for inter-injection and intra-day precisions and below 9% for inter-day precisions. Zwickel et al. calculated intra- and inter-day precisions varying from 3.6 to 9.2% and 4.0 to 10.7% in tomato juice (Zwickel et al., 2016). In contrast to this, a recently published dilute and shoot approach revealed intra-day precisions varying from 9 to 34% at low concentrations and from 3 to 16% at high concentrations in tomato sauce. In wheat flour, intra-day precisions ranged from 11 to 83% at low concentrations and from 3 to 11% at high concentrations (Puntscher et al., 2018).

### Analysis of Infant Food Samples

In our survey, cereal based infant products were frequently contaminated with *Alternaria* mycotoxins, especially AME, TEN, and TA. While the level of contamination of AME and TEN is mainly rather low, high variations in the content of TA were observed with a mean content of 50.2 μg/kg and a median content of 22.0 μg/kg. The variations were detected not only among different grain varieties but also within one grain variety. Grain products such as oat flakes, wheat flour, rye flour, and maize grit have been analyzed already by Asam et al., who found a median content of TA of 16 μg/kg (Asam et al., 2012). In a subsequent study, Asam et al. quantified the toxin in various infant cereal products (Asam and Rychlik, 2013). The products comprising of wheat, oats, rye, spelt, maize, and barley contained TA in amounts of 8–30 μg/kg. Four different millet products were contaminated with high amounts of TA ranging from 130 to 1200 μg/kg (Asam and Rychlik, 2013). In our present survey, two millet infant cereals were analyzed and contained 43.6 and 221 μg/kg of TA. Two of the multi-grain products contained millet flour and were contaminated with 66.0 and 140 μg/kg of TA. In total, none of the analyzed infant food samples in our survey reached the high contamination of millet with TA of up to 1200 μg/kg, which were determined by Asam and Rychlik (2013). It has to be noted that we undertook all effort to collect more millet based infant food samples, but in contrast to 2012 the availability of this product group was very limited. However, as far as it can be deduced from the analyzed samples, the manufactures obviously optimized their quality control and none of the products exceeded the warning limit of 500 μg/kg, which is beneficial for the consumer’s health. As the multi-grain products contained various grains, millet cannot be identified as the sole source of TA contamination.

In 2012, Scott et al. also quantified AOH and AME in various infant foods from Canada. In their survey, single grain as well as multi-grain products were analyzed and revealed 0.5–4.4 μg/kg of AOH and 0.5–2.0 μg/kg of AME (Scott et al., 2012). For Europe, the EFSA assessed the dietary exposure of the European population to *Alternaria* mycotoxins. The mean contamination of wheat grain, spelt grain, and oats were 0.3–39.7 μg/kg of AOH, 0.03–7.1 μg/kg of AME, 0.03–3.8 μg/kg of TEN, and 2.6–168.7 μg/kg of TA (Arcella et al., 2016). In a recently published survey, nine wheat flours from the Austrian market were analyzed for free and modified *Alternaria* mycotoxins. No *Alternaria* mycotoxin could be detected above the respective limits of quantification (Puntscher et al., 2018). In contrast to this, AOH, AME, TEN, ATX I, and TA were detected in two out of four wheat based cereal samples in our study.

A previous survey by our group analyzed various cereal flours for their content of ATX I, ALTP, ATX II, AOH, AME, and TEN (Liu and Rychlik, 2015). ATX I was determined in three different flours with contents of 2.4–4.7 μg/kg. ALTP was determined in one sample (0.87 μg/kg). AOH, AME, and TEN were quantified in contents ranging from 1.1 to 23, 0.31 to 0.34, and 1.6 to 6.0 μg/kg.

Apart from the cereal products we analyzed four different purees in jars, one tomato sauce and one tomato soup. The results of the tomato sauce were striking, because it contained up to 54.2 μg/kg AOH, 7.56 μg/kg AME, and 505 μg/kg TA. When comparing these unexpectedly high contents of mycotoxins to data from the literature, similarly high concentrations amounting to 25 μg/kg of AOH and 5.3 μg/kg of AME have been reported in a triple concentrated tomato paste (Asam et al., 2011a). Analyzing TA in various tomato products, Asam et al. detected 363 to 909 μg/kg of TA in tomato paste (Asam et al., 2011b). The tomato paste was triple concentrated and, therefore, the raw material could be estimated to have contained 120 to 300 μg/kg. The tomato sauce in our survey is ready to use and consists of 65% of tomatoes as well as 13% of concentrated tomato paste. Therefore, it can be assumed that the concentrated tomato paste might be the reason for the high mycotoxin contamination in the final tomato sauce product. In a recent assessment, the EFSA estimated the chronic dietary exposure of humans to *Alternaria* mycotoxins (Arcella et al., 2016). Lower and upper bound mean concentrations ranged from 0.0 to 5.7 μg/kg of AOH, from 0.0 to 1.0 μg/kg of AME, from 0.0 to 3.9 μg/kg of TEN, and from 34 to 54 μg/kg of TA. Tomato puree contained up to 6.5 μg/kg AOH, 1.9 μg/kg AME, 3.3 μg/kg TEN, and 113 μg/kg TA (EFSA, 2011). These data are well in line with those of our previously analyzed puree infant food in jars that contained TA in amounts ranging from 1.0 up to 78 μg/kg. Tomato soup contained 25 μg/kg of TA (Asam and Rychlik, 2013). The TA content of 78 μg/kg in banana and cherry jars from 2012 (Asam and Rychlik, 2013) was not reached in this study, as the TA content in our banana and cherry jar was only 4.51 μg/kg.

In a recently published survey (Puntscher et al., 2018), various tomato sauces (*n* = 12) were analyzed for free and modified *Alternaria* mycotoxins. AOH was detected in two samples above the LOQ (1.4 and 20.2 μg/kg). AME and TEN were determined in one sample and the content was 4.0 and 0.6 μg/kg, respectively. TA was quantified in eight samples with contents ranging from 42 to 323 μg/kg (Puntscher et al., 2018). Although the AOH content of 20.2 μg/kg in one of the tomato sauces was high, the tomato sauce for children in our survey exceeded this value by a factor of two.

From the Swiss market, Noser et al. analyzed 85 tomatoes and tomato products and reported even higher toxin contents. One tomato puree contained 790 μg/kg TA, 30 μg/kg AOH, 8 μg/kg AME, and 2 μg/kg TEN. In tomato sauces and soups 4–144 μg/kg of TA, 4–10 μg/kg of AOH, and 1–4 μg/kg of AME were determined. However, the group did not analyze tomato products which are intended for infant consumption (Noser et al., 2011).

Walravens et al. analyzed 28 tomato sauces and determined AOH in contents up to 41.6 μg/kg, AME up to 3.8 μg/kg, and TA up to 330.6 μg/kg, which is well in line with our findings. Tomato concentrate contained up to 31.0 μg/kg AOH, 6.10 μg/kg AME, and 174.3 μg/kg TA (Walravens et al., 2016).

### Risk Evaluation

A solid risk assessment on *Alternaria* mycotoxins is still not feasible, as toxicological and occurrence data on the toxins are insufficient. Therefore, the TTC approach was applied for AOH, AME, TEN, and TA. For TEN and TA, the TTC level was set to 1500 ng/kg body weight per day (EFSA, 2011). In 2016, Rychlik et al. evaluated the exposure and risk to infants based on the consumption of millet based infant food (Rychlik et al., 2016). In their study, millet based infant food products were contaminated with TA with contents up to 1200 μg/kg. Rychlik et al. calculated an intake of TA of 3670 ng/kg body weight upon consumption of the highly contaminated millet product. A maximum limit of TA in infant food products of 500 μg/kg was claimed so that the daily exposure of infants would fall below the TTC of 1500 ng/kg body weight per day for TA (EFSA, 2011; Rychlik et al., 2016). In the study presented here, all cereal based infant food samples contained TA below 500 μg/kg and, therefore, the products comply with this limit set by Rychlik et al. (2016).

Due to their genotoxicity, the TTC levels for AOH and AME were set to 2.5 ng/kg body weight per day (EFSA, 2011). One spelt product in our survey contained 7.17 μg/kg AOH. Considering an infant’s weight of 7 kg and the size of a portion of 18 g, as suggested by the manufacturer, the intake would be 18.4 ng/kg body weight. This exceeds the TTC value by a factor of around seven. Therefore, a risk to the health of the infant cannot be excluded. One millet, and two multi-grain samples contained 1.01, 3.40, and 1.37 μg/kg of AOH, respectively. Calculating the intake of AOH based on the portions size of 20 to 25 g, the intakes also exceed the TTC value of 2.5 ng/kg. Considering the mean content of AOH of 0.89 μg/kg from our limited survey, the respective intake would not exceed the TTC level of 2.5 ng/kg body weight per day (EFSA, 2011). Moreover, the determined contents of AME and TEN in the cereal based infant food products were rather low and cannot be considered as a risk to the health of infants.

As further infant foods, four purees in jars and two tomato products were analyzed for mycotoxin contamination. Especially the tomato sauce contained high amounts of AOH, AME, TEN, and TA. Considering the size of a portion of 50 g and an infant’s weight of 10 kg at the age of 12 months, the intake exceeds the TTC levels of AOH and AME many times over. Therefore, for this individual product, a risk to the health of infants cannot be excluded (EFSA, 2011) and we called the manufacturer’s attention to this contamination. The median contents of the purees and tomato products were 0.32 μg/kg for AOH, 0.14 μg/kg for AME and 2.51 μg/kg for TA. On the basis of these calculations, the consumption of the infant food products do not constitute a risk to the health of the infants on average (EFSA, 2011).

Not only the free forms of the *Alternaria* mycotoxins attract the interest in quantitative analysis, but also the modified forms of AOH and AME are more frequently analyzed. Puntscher et al. quantitated the AOH-9-glucoside and the AOH-3-sulfate and AME-3-sulfate in food samples above the respective limits of quantification for the first time. Due to these modified forms of *Alternaria* mycotoxins, human health might be much more endangered than assumed and should be considered for tolerable daily intake determination (Puntscher et al., 2018). Further studies are underway to include also these toxins into our validated method.

## Conclusion

A LC-MS/MS method for the simultaneous determination of six *Alternaria* mycotoxins was developed and the extraction and purification of mycotoxins was optimized. The method was validated successfully according to Vogelgesang and Hädrich (1998). The method validation was performed in starch and tomato matrix and resulted in low LODs and LOQs and good recoveries for all analytes. Good precisions confirmed stability and robustness of the method.

Various cereal based infant foods, jars containing vegetables and fruits and tomato based products for infants were analyzed for *Alternaria* mycotoxins. Studies on infant foods are rare and so far were limited to certain toxins (Scott et al., 2012; Asam and Rychlik, 2013). Our study included six toxins in the analysis of infant foods for the first time. The analyses resulted in partly high contaminations of the infant food products and risks to the health of infants cannot be excluded. These new and unexpected results show that more infant food products should be analyzed to determine mycotoxin contamination and to perform a proper risk assessment with special regards to infants. Moreover, the manufacturers should be made aware of the necessity to screen their products more thoroughly for these toxins and take the appropriate measures to reduce their contents.

## Author Contributions

SA, MG, and MR designed the experiments. KG optimized the mycotoxin extraction and purification and performed the method validation for starch matrix. RK optimized analyte purification by solid phase extraction. AM performed the method validation for tomato matrix. EB analyzed the infant food samples. MG and SA wrote the manuscript. MR revised the manuscript. All authors contributed to the revision of the manuscript.

## Conflict of Interest Statement

The authors declare that the research was conducted in the absence of any commercial or financial relationships that could be construed as a potential conflict of interest. The reviewer BŠ declared a past co-authorship with one of the authors MR to the handling editor.
